# Aberrant Default-Mode Functional and Structural Connectivity in Heroin-Dependent Individuals

**DOI:** 10.1371/journal.pone.0120861

**Published:** 2015-04-10

**Authors:** Xiaofen Ma, Yingwei Qiu, Junzhang Tian, Jinhui Wang, Shumei Li, Wenfeng Zhan, Tianyue Wang, Shaoqing Zeng, Guihua Jiang, Yikai Xu

**Affiliations:** 1 Department of Medical Imaging Center, Nanfang Hospital, Southern Medial University, Guangzhou, PR China; 2 Department of Medical Imaging, Guangdong No. 2 Provincial People’s Hospital, Guangzhou, PR China; 3 Center for Cognition and Brain Disorders, Hangzhou Normal University, Hangzhou, PR China; 4 Zhejiang Key Laboratory for Research in Assessment of Cognitive Impairments, Hangzhou, PR China; University of Modena and Reggio Emilia, ITALY

## Abstract

**Background:**

Little is known about connectivity within the default mode network (DMN) in heroin-dependent individuals (HDIs). In the current study, diffusion-tensor imaging (DTI) and resting-state functional MRI (rs-fMRI) were combined to investigate both structural and functional connectivity within the DMN in HDIs.

**Methods:**

Fourteen HDIs and 14 controls participated in the study. Structural (path length, tracts count, (fractional anisotropy) FA and (mean diffusivity) MD derived from DTI tractography)and functional (temporal correlation coefficient derived from rs-fMRI) DMN connectivity changes were examined in HDIs. Pearson correlation analysis was performed to compare the structural/functional indices and duration of heroin use/Iowa gambling task(IGT) performance in HDIs.

**Results:**

HDIs had lower FA and higher MD in the tract connecting the posterior cingulate cortex/precuneus (PCC/PCUN) to right parahippocampal gyrus (PHG), compared to the controls. HDIs also had decreased FA and track count in the tract connecting the PCC/PCUN and medial prefrontal cortex (MPFC), as well as decreased functional connectivity between the PCC/PCUN and bilateral PHG and MPFC, compared to controls. FA values for the tract connecting PCC/PCUN to the right PHG and connecting PCC/PCUN to the MPFC were negatively correlated to the duration of heroin use. The temporal correlation coefficients between the PCC/PCUN and the MPFC, and the FA values for the tract connecting the PCC/PCUN to the MPFC were positively correlated to IGT performance in HDIs.

**Conclusions:**

Structural and functional connectivity within the DMN are both disturbed in HDIs. This disturbance progresses as duration of heroin use increases and is related to deficits in decision making in HDIs.

## Introduction

Compulsive drug seeking and use, despite serious negative consequences, characterizes drug addiction [1]. Heroin is the most commonly abused illicit substance in China. Indeed, approximately 83.3% of illicit drug addicts in China use heroin [2]. In the United States, there are over 1.2 million “occasional” heroin users and over 200,000 people who can be classified as addicted to the drug, according to the National Survey on Drug Use and Health. However, the exact neurobiologic mechanisms underlying heroin addiction are still not fully understood. Therefore, the investigation of heroin dependent individuals (HDIs) has great potential to improve the understanding of disease pathophysiology.

The default mode network (DMN) is a collection of brain regions that reliably deactivate during goal-directed behaviors and is more active at baseline, or at a so-called resting condition [3,4]. The default mode areas are commonly thought to include the posterior cingulate cortex / precuneus in the medial parietal lobe, the medial prefrontal cortex, and the lateral parietal cortex [3,5]. The DMN is believed to support important core processes such as implicit learning, autobiographical memory retrieval, prospection, monitoring executive control and other internally focused thought processes [6], many of which are reported to show heroin addict-related alterations[7,8]. This implies the significance of the DMN in understanding the pathophysiology of heroin addiction. In the current study, we thus hypothesized that the DMN would exhibit abnormal functional and structural organization induced by drug heroin addiction, particularly in interregional connectivity within the DMN. This is based on the fact that accumulating evidence suggests that interregional neural synchronization underpins a diverse range of cognitive domains, such as memory retrieval [9]and cognitive control[10]. However, very little is currently known about heroin-related DMN functional connectivity disorder and even less is known about structural changes that underlie the DMN functional disorder.

Imaging plays an important role in detecting structural and functional abnormalities in the brain. Diffusion tensor imaging (DTI) findings have established both axonal disruption and widespread demyelination in HDIs, which progresses with the duration of heroin use [11, 12]. DTI fiber tractography is a direct way of depicting the structural connectivity of the brain network[13,14]. This approach can be used to estimate the routes taken by the fiber pathways that connect different brain regions in humans. DTI fiber tractography has been proven to be an effective way to image the brain in individuals with various mental disorders [15,16]. However, to the best of our knowledge, no study has used DTI tractography to examine white matter within the DMN of HDIs. Resting-state functional connectivity, assessed by the correlation of spontaneous fluctuations of blood oxygen level-dependent(BOLD) signals in different regions of the ‘‘resting” brain, is believed to provide a measure of the functional organization of the brain. A previous studies on resting-state functional connectivity has demonstrated that functional integrity is abnormal within the DMN of HDIs [17]; however, the relationship between DMN structure and function in HDIs has not yet been elucidated. Examining heroin-related DMN changes is important not only for understanding how structural brain changes disable key functional brain circuits but also for understanding the impairment of cognitive processes promoted by the DMN.

The Iowa Gambling Task (IGT)was specifically developed to assess and quantify decision-making abilities by simulating real-life decision making under ambiguous conditions [18]. Converging evidence indicates that decision-making performance is impaired in HDIs [19–22]. The IGT was performed shortly after MR imaging to assess decision making in participants under uncertain conditions.

In the current study, it is hypothesized that both structural and functional connectivity within the DMN is disturbed in HDIs. DTI tractography was combined with resting state fMRI (rs-fMRI) to investigate the structural and functional connectivity within the DMN in HDIs. The purpose of this study was to identify structural and functional connectivity changes within the DMN in HDIs and to investigate whether any changes in fiber tracts and temporal correlation coefficients are related to duration of heroin use and/or to decision-making deficits in HDIs.

## Materials and Methods

### Subjects and data acquisition

Fourteen male heroin-dependent individuals (mean age = 37.86±7.70 years) were recruited from the Addiction Medicine Division of the Guangdong No. 2 Provincial People’s Hospital. Each HDI was screened and a diagnosis of opiate dependence was confirmed using the Structured Clinical Interview (SCID-I) from the Statistical Manual of Mental Disorders, Fourth Edition (DSM-IV). Urine tests positive for heroin use were acquired from each HDI prior to enrolling in the treatment program. The following exclusion criteria were set in order to avoid confounding factors from comorbidities with heroin use: no dependence on other psychoactive drugs and no history of drug use other than heroin and nicotine (smoking). Fourteen healthy male volunteers were recruited as controls (mean age = 36.36±6.10 years) according to their medical record and/or their statement. Other exclusion criteria for both groups were as follows: None of the subjects were taking prescription drugs within 1 week that affected the central nervous system and had a history of neurological illness or head injury. No diagnosis of either schizophrenia or affective disorder and other mental disorder disease, no abnormality on the MRI scan, no additional substance abuse/dependence diagnosis, and no contraindications for MRI scanning. All subjects were right-handed, according to self-reports. Table 1 lists the demographic details for all subjects.

**Table 1:** Demographic and Clinical Characteristics of HDI and Control Groups.

*Characteristic*	*HDI group (n = 14) (mean ±SD)*	*Control group (n = 14) (mean ±SD)*
Age (y)	37.86±7.70	36.36±6.10
Gender	14M/0F	14M/0F
Education (y)	10.07±3.25	10.86±3.01
Nicotine (no. of cigarettes/d)	19.64±9.50	16.79±7.75
Heroin (y)	8.79±4.31	N/A
Mean dosage (g/d)	0.6±0.4 (rang: 0.1~1.2)	N/A
Onset of heroin use (y)	24.41±3.24	N/A
Routes of administration	Injected(9); inhaled by snorting or sniffing (7)	N/A
IGT scores	-3.43±5.626 (rang:-14~6 n = 14)	6.29±6.603 (rang:-6~20 n = 14)

There was no significant difference between the groups in age (t_(26)_ = -0.571,p = 0.573), educational level (t_(26)_ = 0.664,p = 0.512), nicotine consumption (t_(26)_ = -0.872,p = 0.391), sex composition between heroin-dependent and control groups. There was significant difference between the groups in IGT performance (t_(26)_ = 4.190,p<0.001).

This study was approved by the Research Ethics Review Board of the Institute of Mental Health of the Southern Medical University in Guangzhou, China. Written informed consent was obtained from each subject.

### Behavioral Measures

The original card version of the IGT was used to evaluate decision-making performance. The IGT was administered according to the procedure described by the Iowa group [18]. In the task, the “bad” decks of cards yielded higher immediate rewards along with unpredictable and larger delayed punishments, while the “good” decks yielded lower immediate gains along with unexpected and smaller future losses. Quality of decision making was measured by the overall net score, which was obtained by subtracting the number of cards selected from the “bad” decks from the number of cards selected from the “good” decks [(C+D)−(A+B)]. Participants selected, on average, approximately 20 cards/min. This task has been shown to differentiate between patients with frontal cortical lesions and healthy controls [23, 24], as well as between drug users and controls [20].

### MRI Scanning

MRI data were obtained using a Philips Achieva 1.5T Nova dual MR scanner in the Department of Medical Imaging, Guang Dong No.2 Provincial People’s Hospital. Each subject was in a supine position with the head snugly fixed by a belt and foam pads. During resting state fMRI scanning, subjects were instructed to close their eyes, keep as still as possible and not to think of anything systematically or fall asleep. The scanning sessions included (i) localization, (ii) T1 anatomy (22 axial slices, thickness/gap = 4.5/0 mm, in-plane resolution = 256×256, FOV (field of view) = 230×230 mm^2^, TR (repetition time) = 4000 ms, TE (echo time) = 29 ms), (iii) rs-fMRI session (22 axial slices, thickness/gap = 4.5/0 mm, in-plane resolution = 64×64, FOV = 230×230 mm^2^, TR = 2000 ms, TE = 50 ms, flip angle = 90°, and 240 volumes (8 minutes) (the slices were approximately along the AC-PC line and covered about −30 to 60 in the IS direction), (iv) 3D-T1 session covering the whole brain (132 axial slices, TR = 25 ms, TE = 4.1 ms, thickness = 1.0 mm, no gap, in-plane resolution = 231×232, FOV = 230×230 mm^2^, flip angle = 30), (V)DTI data were acquired in 32 diffusion gradient directions (b = 800 s/mm2 along 32 non-collinear directions) plus a b = 0 reference image using a single shot spin echo planar sequence (TR = 10793 ms, TE = 62 ms, FOV = 230×230 mm^2^, matrix = 128×128, slice thickness = 2mm, with no slice gap. voxel size = 2×2×2 mm^3^). After the scans, each participant was asked questions to verify the degree of cooperation.

### Data preprocessing

Prior to preprocessing, all MRI images were reviewed by a neuroradiologist to exclude participants with signs of vascular accidents or other abnormalities. No participants were excluded. All 32 diffusion-weighted DTI images from each subject were first registered to the B0 image (b = 0s/mm2),using SPM 8(http://www.fil.ion.ucl.ac.uk/spm), and then corrected for the difference in spatial distortion due to eddy currents using FMRIB’s Diffusion Toolbox (FDTv2.0)as implemented in FMRIB’s Software Library (FSL v4.1; www.fmrib.ox.ac.uk/fsl) [25].

Preprocessing of fMRI images was performed using the SPM8 software package. The first 10 images were excluded to take into account instability in the magnetic field. The remaining 230 consecutive volumes were used for data analysis. After adjusting for slice-timing offsets and head motion, the functional images were spatially normalized to standard stereotaxic coordinates, according to the Montreal Neurological Institute (MNI), resampled into a voxel size of 3×3×3 mm^3^. Any data showing excessive head motion (>1mm of translation or >1°of rotation in any direction) were excluded. Moreover, we further employed a 24-parameter model [26] to control head motion effect, which is demonstrated to be an efficient strategy in attenuating residual head motion effects [27]. Using the 24-parameter head motion model, we just found similar results, indicating the residual head motion effect is negligible for the current dataset. (S1 Fig)The normalized data were then spatially smoothed by convolution with an isotropic Gaussian kernel (FWHW = 8 mm).

### DMN extraction

In order to extract the regions of interest (ROIs) within the DMN, spatial independent component analysis (ICA) was conducted to decompose the smoothed data from each individual in the control and HDI groups into 32 and 36 independent components(ICs), respectively via the Infomax algorithm using Group ICA of the fMRI Toolbox (GIFT, http://icatb.sourceforge.net/, Version 2.0d) [28]. The number of ICs was determined by a dimension estimation using the minimum description length (MDL) criterion [29]. Controls and HDIs in this study were estimated separately to avoid mixing the specific resting-state network pattern from each group [30,31]. For each IC, the waveform corresponds to the time course of a specific pattern of coherent brain activity and the intensity of this pattern across the voxels is expressed by the associated spatial map [5,32]. The default mode component, including the bilateral inferior parietal lobe (IPL), inferior temporal gyrus (ITG), parahippocampalgyrus (PHG), posterior cingulated cortex/precuneus(PCC/PCUN) and medial prefrontal cortex (MPFC), was then selected using the GIFT toolbox in which all components were spatially correlated with the DMN spatial template. This DMN template was provided by Dr. Liao (Center for Cognition and Brain Disorders and the Affiliated Hospital, Hangzhou Normal University, Hangzhou, China) and has been used in previous studies [33]. The intensity values in each spatial map were converted to z values to display the voxels that contributed most strongly to a particular spatial IC. The z-values here reflect the degree to which the time courses of a given voxel correlate to the time courses of each special IC[5, 32]. Group maps were obtained using one-sample t-tests using a threshold of P<0.01 false discovery rate (FDR)-corrected for multiple comparisons. Group comparisons were done using two sample t-tests with athreshold of P<0.05 FDR-corrected for multiple comparisons. Regions of interest (ROIs) for the PCC/PCUN, MPFC, and bilateral IPL, PHG, and ITG were selected from the control group DMN map using the xjViewtoolbox [15,16] (see Table 2 for details of these ROIs). These eight ROIs were used for subsequent analyses in both the HDI and the control groups.

**Table 2:** Details on the brain regions in the DMN map (P<0.01, FDR corrected) from the control and HDIs.

*Anatomical region*	*Hemisphere*	*MNI coordinates (x*,*y*,*z)*	*Brodmann’s area*	*Cluster size (voxels)*	*T value*
**Controls**					
PCC/PCUN	L/R	6,-60,30	7,30,31	4286	20.70
IPL	L	-51,-63,36	19,39,40	504	13.21
	R	45,-63,30	39,40	921	13.43
PHG	L	-27,-23,-15	35,36	49	6.92
	R	27,-23,-17	35,36	52	7.13
ITG	L	-42,12,-39	20,21,38	997	12.79
	R	42,21,-24	20,21,38	1581	14.49
MPFC	L/R	6,48,12	8,9,10,11,32	4908	25.38
**HDIs**					
PCC/PCUN	L/R	-3,-46,30	7,30,31	1968	10.26
IPL	L	-48,-66,21	19,39,40	448	8.35
	R	39,-66,30	39,40	801	11.52
PHG	L	-20,-21,-24	35,36	82	7.25
	R	25,-16,-24	35,36	105	8.42
ITG	L	-55,-2,-24	20,21,38	744	7.33
	R	60,-6,-24	20,21,38	1096	8.36
MPFC	L/R	9,45,9	8,9,10,11,32	3019	13.26
**HDI〈 Control**					
Middle temporal gyrus	R	57,-18,-15	20,21,38	215	-7.25
OFC	L/R	6,48,-21	11	60	-9.08
PHG	L	-9,-42,-3	30	106	-4.92
Superior frontal gyrus	L/R	18,66,18	8,9,10	916	-7.20
IPL	R	57,-48,27	39,40	156	-6.70
IPL	L	-51,-60,39	39,40	137	-6.81
Paracentral lobule	L/R	0,-21,51	5,6,7,31	457	-5.69

### Structural connectivity within the DMN

Whole-brain fiber tracking was performed in the DTI native space of each subject using the Diffusion ToolkitandTrackVis software(http://www.trackvis.org/)with an interpolated streamline propagational gorithm [34]. Path tracing proceeded until either the FA fell below 0.15 or the minimum angle between the current and the previous path segment was higher than 35° [15,16,34]. Short fibers, less than 20 mm, and obvious false paths were discarded after whole fiber tracking. The remaining tracts were highly congruent with the DTI tractography atlas [35,36].

Since the eight ROIs within the DMN were derived from the normalized MNI space, the inverse transformation of the spatial normalization was applied to acquire the ROIs in the native space of the DTI [14,15, 36]. More specifically, the inverse transformation (*T* ^-1^) was applied to the eight ROIs in the normalized MNI, resulting in subject-specific ROIs in the DTI native space.

The ROIs were dilated 2–3 mm into the white matter to ensure that they were in contact with the fibers [16]. Fiber bundles connecting each pair of ROIs were then extracted from the total collection of brain fibers as follows. First, an initial ROI was selected and the tracts that reached the first ROI were chosen from all fibers. Second, another ROI was retrieved from the rest of the ROIs. Only those tracts that reached the second ROI were chosen from the resulting tracts in the previous step. Finally, fibers that were an atomically implausible were visually identified and removed. The remaining fiber bundles that connected each pair of ROIs were prepared for subsequent analyses. In the present study, four basic indices of fiber connectivity, including path length, tract count, mean fractional anisotropy (FA) and mean diffusivity (MD) of each fiber pathway within DMN, as obtained from TrackVis [15,16], were used for the structural connectivity analysis to determine whether there were any abnormalities in HDIs compared to the healthy controls.

### Functional connectivity within the DMN

Interregional functional connectivity between each pair of ROIs within the DMN were estimated using the Pearson correlation coefficient. The time series for each ROI was preprocessed as follows. First, the functional ROIs were inversely transformed to the native space in the same way as the DTI for the aforementioned eight ROIs within the DMN. Second, to extract the time series for cerebrospinal fluid (CSF) and white matter (WM), each individual’s T1 weighted anatomical images were segmented using SPM8, with the threshold of the segmented probability images setting at 80% [15,16]. The segmented CSF and WM were then co-registered to the individual’s B0 images (b = 0 s/mm2) to create subject-specific CSF and WM templates. Third, the time series were extracted from each ROI in the DTI native space and several sources of spurious variance were then removed by linear regression, including six head motion parameters, as well as average signals from the subject-specific CSF, WM and whole brain masks. Fluctuations unlikely to be involved in specific regional correlation were removed via this regression. Fourth, the residuals time series were band filtered (0.01–0.08 Hz) and the functional connectivity of brain regions within the DMN was computed by calculating the temporal correlation coefficients between the residuals time series in each pair of regions.

### Statistical analysis

Structural and functional connectivity for each pair of ROIs were compared between the HDI and control groups. Specifically, structural connectivity (including path length, tract count, fractional anisotropy and mean diffusivity) and functional connectivity (temporal correlation coefficient [after Fisher-z translation]) were analyzed using two sample t-tests (P<0.05, corrected for multiple comparison using the Bonferroni correction for the number of tracts that showed structure in all subjects. Three tracts [fiber tracks that connected the PCC/PCUN with the MPFC and bilateral PHG]were considered in the present study).

Structural/functional connectivity indices that showed differences between HDIs and controls were compared with the duration of heroin use and IGT performances, using the Pearson correlation analysis, to investigate the effect of duration of heroin abuse on structural and functional connectivity within the DMN and the relationship between the IGT performances and DMN connectivity indices.

Correlation analysis was performed using SPSS 13.0 (SPSS, version 13.0; SPSS, Chicago, IL, USA). The threshold was set at a significance level of P<0.05, corrected for multiple comparisons using the Bonferroni correction for the number of tracts that showed either different structural or functional connectivity between HDI and controls(three tracts were considered in the Bonferroni correction in this study[fiber tracks that connected the PCC/PCUN with the MPFC and bilateral PHG]) [14].

## Results

### Spatial pattern of the DMN in each group

**Fig 1:**
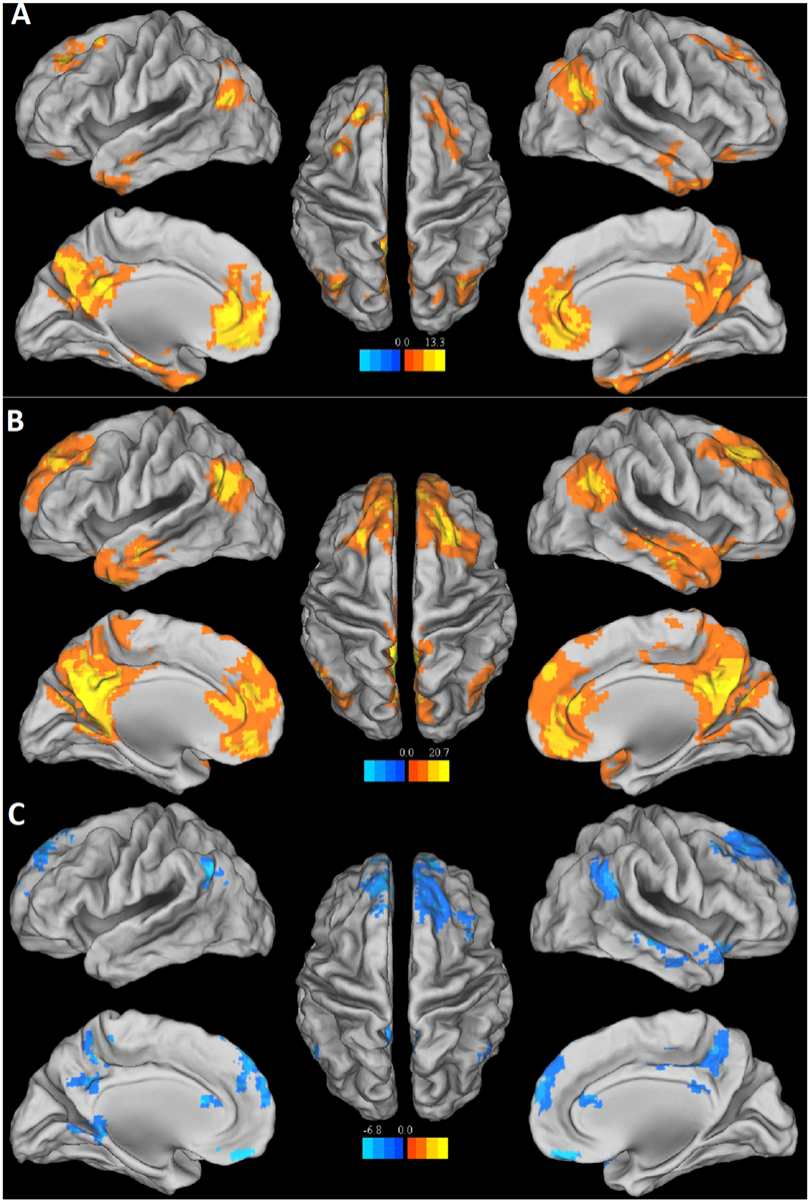
Group-level analyses of the DMN. A: DMN in the HDI (T> 3.16, P < 0.01, FDR corrected). B: DMN in the Control (T>4.23, P < 0.01, FDR corrected). C: Contrast between the DMN in HDI and controls (| T | >2.7, P < 0.05, FDR corrected). T values, cluster size, and Talairach coordinates of all significant clusters are reported in Table 2.

The random-effect analysis of the single-subject DMN maps revealed a typical spatial pattern of the DMN in both groups(P<0.01, FDR corrected) (Fig 1). The DMN pattern in the controls (Fig 1B and Table 2) showed functional connectivity among the PCC/PCUN, the MPFC and the bilateral IPL, PHG, and ITG. The DMN pattern of the HDI group (Fig 1A and Table 2)largely included the same brain regions as in the controls, but with specific differences in the connectivity strength (Fig 1B and Table 2). In particular, the HDI group showed decreased DMN connectivity in the OFC, the bilateral IPL, the bilateral superior frontal gyrus, the bilateral paracentral lobule, the left PHG and the right middle temporal gyrus. (Fig 1C and Table 2)

To avoid bias, we also analyzed relevant results by using a single ICA analysis with all the 28 patients in one group. Similar results were found. Alternatively, we also used the anatomical template to determine the default mode network for the both groups. Again, comparable results were obtained with those reported in the main text. These findings suggest that our results were robust against the methods used to identify the default mode network. (S2 Fig)

### Structural connectivity within the DMN

**Fig 2:**
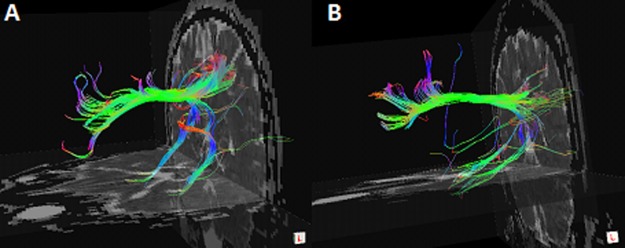
Example of DTI fiber tractography on one control (A) and one HDI (B). Only three fiber bundles connecting the PCC/PCUN and MPFC, bilateral PHGs were detected in all the subjects. The color-coding of the obtained fibers is based on the standard RGB (Red, Green, Blue) code applied to the vector at every segment of each fiber. Red indicates the medio-lateral plane. Green indicates the dorsoventral orientation. Blue indicates the rostro-caudal direction. DTI = diffusion tensor imaging; HDI = heroin dependent individual; PCC/PCUN = posterior cingulated cortex/precuneus; MPFC = medial prefrontal cortex; PHG = parahippocampalgyrus.

**Fig 3:**
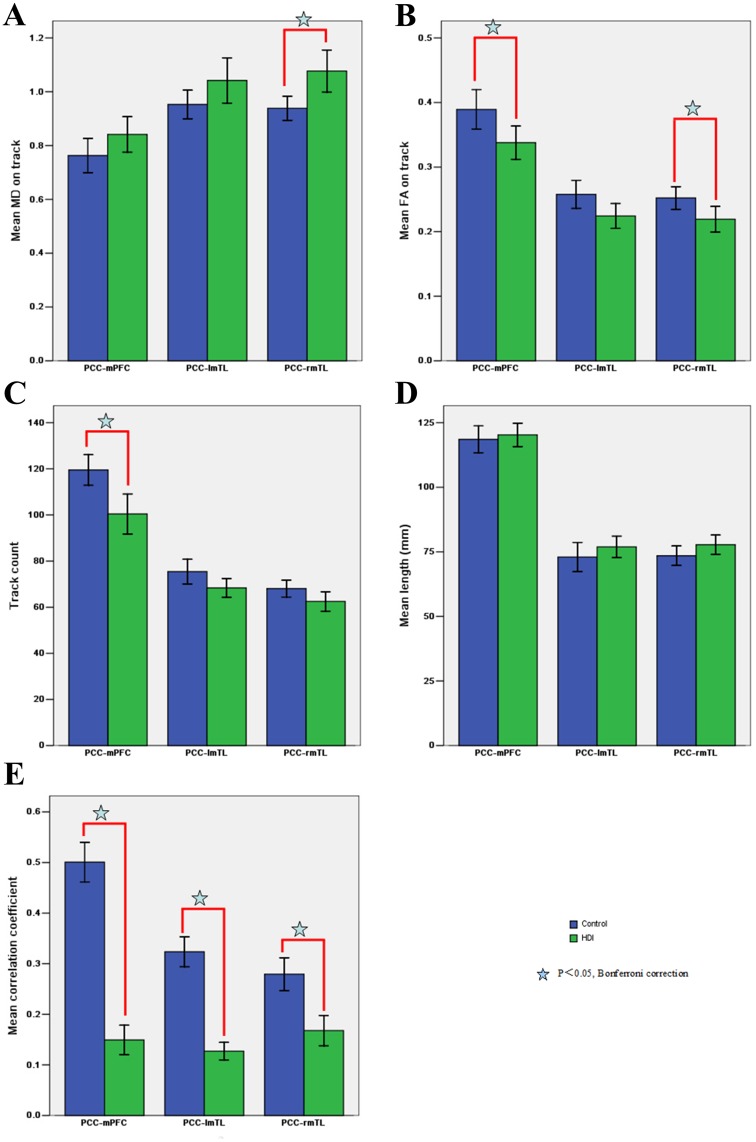
Comparison of structural and functional connectivity between HDIs and Controls. Compared to the Controls, HDIs show both decreased FA and increased MD between the PCC/PCUN to right PHG, decreased FA and track count between the PCC/PCUN and MPFC, and decreased functional connectivity between the PCC/PCUN and bilateral PHG, and MPFC. For each connection, differences are setting at the significant level of corrected P<0.05(marked with star symbol) with Bonferroni correction. Error bars represent the standard deviation of the measurements. HDI = heroin-dependent individuals; PCC/PCUN = posterior cingulate cortex/precuneus; PHG = parahippocampalgyrus; MPFC = medial prefrontal cortex; FA = fractional anisotropy; MD = mean diffusivity.

Two examples of fiber tracts connecting the PCC/PCUN to the mPFC and to the bilateral mTLs are shown in Fig 2. The cingulum tracts, connecting the PCC/PCUN to mPFC, were detected in all controls and all HDIs. The tracts connecting the PCC/PCUN to bilateral mTLs (both left and right mTL ROIs) were detected in all participants in both groups. The left and right superior frontal-occipital fasciculus, connecting the left and right IPLs to the mPFC, were detected in 5/4 (left/right) out of 14HDIs and 7/7 (left/right) out of 14 controls. The tracts that connected the PCC/PCUN to the left and right ITG were detected in 0/1 (left/right) of 14HDIs and in 0/3 (left/right) of 14 controls. Therefore, only the structural indices of three tracts detected in all subjects were further compared (the fiber bundles located between the PCC/PCUN and the MPFC, bilateral PHG), as in previous studies [15,16]. HDIs had an increased MD (p = 0.009 Fig 3A) and decreased FA (p = 0.039; Fig 3B) in the tract connecting the PCC/PCUN to right PHG and a decreased FA (p = 0.030; Fig 3B) and track count (p = 0.003; Fig 3C)in the tract connecting the PCC/PCUN and MPFC. No differences were found in any structural connectivity indices in the fibers bundles connecting the PCC/PCUN to the left PHG (all P>0.05)(Fig 3).

### Functional Connectivity within the DMN

**Fig 4:**
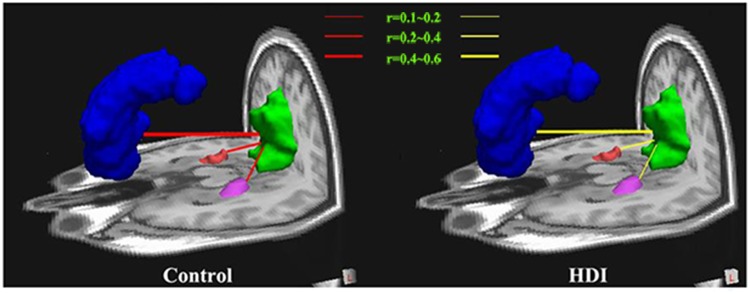
Functional connectivity within the DMN in Controls and HDIs. Averaged temporal correlation coefficient r (Red) across all subjects in the Controls (A) and in the HDIs (B). Compared with Controls, HDIs show decreased temporal correlation coefficients between the PCC/PCUN and the MPFC, as well as between the PCC/PCUN and the bilateral PHG. MPFC is color-coded Blue, PCC/PCUN color-coded Green, the left PHG color-coded Pink, the right PHG color-coded Red. DMN = default mode network; HDI = heroin-dependent individuals. PCC/PCUN = posterior cingulate cortex/precuneus; MPFC = medial prefrontal cortex; PHG = parahippocampalgyrus.

According to the results from the structural connectivity analysis, only three pair-wise functional connectivities (the PCC/PCUN to the MPFC, the left mTLs and the right mTLs) were examined (Fig 4). The temporal correlation coefficients between the PCC/PCUN and the MPFC (p<0.001, t = 15.598) and between the PCC/PCUN and the bilateral PHG (p<0.001, t = 11.419 for right and t = 5.812 for the left) were lower in HDIs than in controls (Fig 3E).

### Pearson correlation results

**Fig 5:**
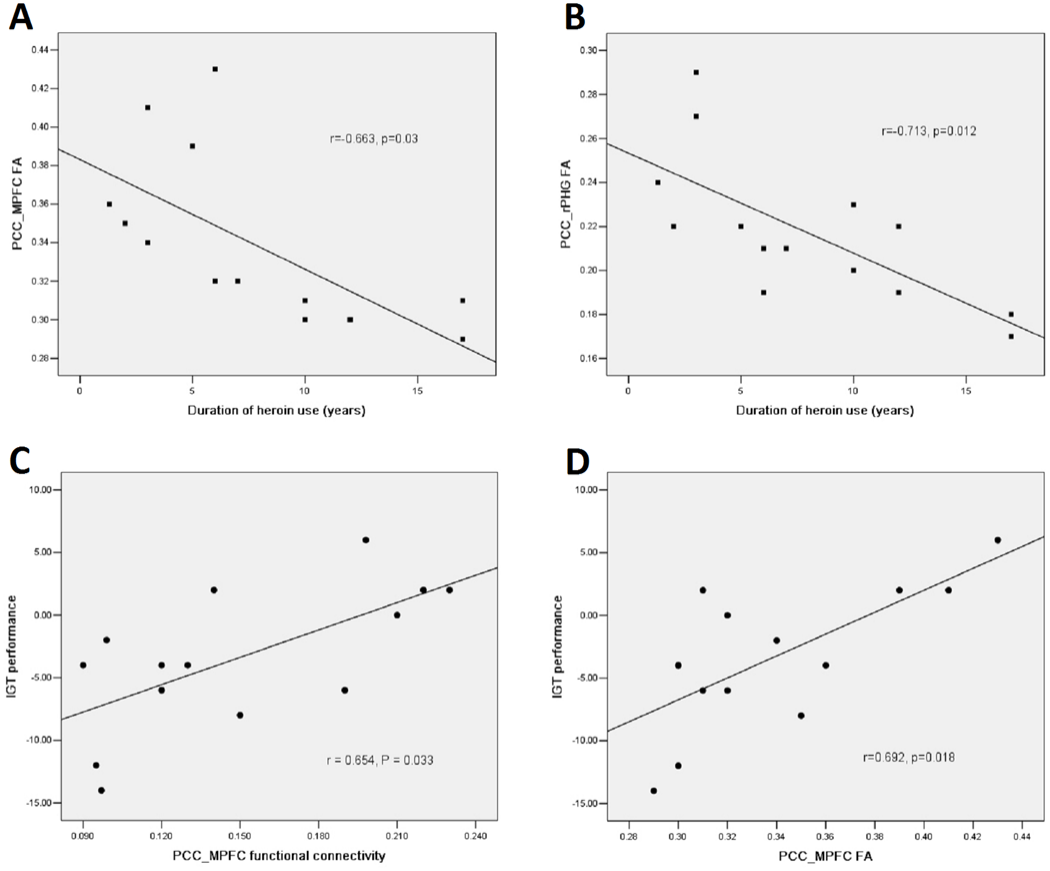
Correlationbetweenthe structural/functional indices and clinical markers of HDIs. Pearson correlation results reveal that in HDIs, the FA values of the tracts connecting PCC/PCUN to the MPFC (A), and connecting PCC/PCUN to the right PHG (B)negatively correlate to the duration of heroin use, the temporal correlation coefficients between PCC/PCUN and the MPFC (C), and the FA values of the tracts connecting PCC/PCUN to the MPFC (D) show positive correlation to the IGT performance (P<0.05, Bonferroni corrected). HDI = heroin dependent individuals; FA = fractional anisotropy; PCC/PCUN = posterior cingulate cortex/precuneus; PHG = parahippocampal gyrus; MPFC = medial prefrontal cortex; IGT = Iowa gambling task.

The FA of the fiber bundle connecting the PCC/PCUN to the MPFC (r = -0.663, p = 0.03) and right PHG (r = -0.713, p = 0.012) was negatively correlated to the duration of heroin use (Fig 5A and 5B) in the HDI group (Fig 5). The temporal correlation coefficients between the PCC/PCUN and the MPFC (r = 0.654, P = 0.033) and the FA values of the tract connecting the PCC/PCUN to the MPFC (r = 0.692, P = 0.018)were positively correlated to the IGT performance in the HDI group (Fig 5C and 5D). The temporal correlation coefficients between the PCC/PCUN and was positively correlated to the IGT performance in the control group (r = 0.714, P = 0.012). There was no significant correlation between other structural and functional indices with the duration of heroin use or IGT performance.

## Discussion

In the present study, the functional (temporal correlation coefficients derived from rs-fMRI)and structural (FA, MD, tract count, and mean length from DTI tractography) connectivity of the DMN was investigated in HDIs. It was discovered that both structural and functional connectivity is disturbed within the DMN of HDIs. In addition, some structural and functional connectivity indices were significantly correlated with the duration of heroin use and IGT performance in HDIs. This study linking connectivity at rest with brain structure and function furthers our understanding of the neuropathophysiology of heroin dependence.

First, we use Independent component analysis (ICA) to observe the functional connectivity at the network level. ICA is a data-driven method, it can extract the DMN and examine the functional connectivity of the entire network [37]. The ICA results of the present study showed that functional connectivity was lower in several brain regions in HDIs, including the OFC, the bilateral IPL, the bilateral superior frontal gyrus, the bilateral paracentral lobule, the left PHG and the right middle temporal gyrus within the DMN (Fig 1C and Table 2). Since the DMN is a core network, which is involved in self-referential mental activity and social cognition [38],the widespread decrease in functional connectivity within the DMN in HDIs in the present study may refiect the cognitive impairments of HDIs. These results are somewhat consistent with those of previous studies of HDIs, using ICA. Ma et al. found increased functional connectivity in the right hippocampus and decreased functional connectivity in right dorsal anterior cingulate cortex and left caudate in the DMN of heroin users, leading to abnormally increased memory processing, but diminished cognitive control related to attention and self-monitoring. They postulated that these changes were likely addiction-related abnormalities [17]. However, no increase in functional connectivity was found in HDIs in the present study. This may be due to the diverse characteristics of the HDIs between the 2 studies. All of the HDIs in the present study were under methadone maintained treatment (MMT), while 9 of the 12 HDIs in the Ma study were in the detoxification phase. MMT itself can affect brain activity and brain connectivity [39,40] and future studies are needed to explore the effect of MMT on the DMN of HDIs. Importantly, the ICA approach used in the current study enabled us to extract brain regions in the DMN as ROIs, without the need of prior anatomical hypotheses to test the functional and structural connectivity across those ROIs.

For investigated the disrupted structural connectivity within DMN in HDIs, the current study mainly focused on the cingulum and the tracts connecting the PCC/PCUN to the bilateral PHG interconnecting the brain regions within the DMN, which were found consistently across all subjects. The tract connecting the PCC/PCUN to right PHG showed decreased FA and increased MD, while the tract connecting the PCC/PCUN to the MPFC showed decreased FA and tract count in HDIs compared with controls. These changes can be attributed to axonal disruption and demyelination in these tracts and can also be explained by the less dense packing of axonal fibers in HDIs. Results of this study support the findings of previous studies that have shown widespread axonal disruption and demyelination in HDIs [11,12]. Furthermore, the present study suggests that a decrease tract number and density may also exist in HDIs. Thus, the combination of FA and the quantitative properties(i.e. tract number and fiber length), may provide better insights into HDIs.

We also found the disturbed functional connectivity within the DMN in HDIs. As we know, functional connectivity is typically interpreted as the temporal synchronization of low-frequency fluctuations arising from spontaneous neuronal activities in distant brain regions [41]. In the present study, the functional connectivity between the PCC/PCUN and MPFC and bilateral PHG was significantly decreased in HDIs. The PCC discerns emotional and self-relevant information; this interacts with the ACC, which integrates emotional information with cognition, and the MPFC, which allows for self-reflection and the regulation of emotion and arousal [42]. The reduced functional connectivity between the PCC/PCUN and MPFC/ACC may be related to the deficits in emotional regulation and cognition. Given that the PHG plays an important role in memory encoding and retrieval [43], the reduced functional connectivity between the PCC and PHG may be related to the impairment of retrieval and memory encoding in HDIs. Reduced functional connectivity between the PCC and PHG is generally observed in older people [44], thus the results of the current study may further support a recent study, which indicated that heroin abuse can accelerate biological aging [45].

In the present study, the finding that the FA values of the tract connecting the PCC/PCUN to the MPFC and the FA values of the tract connecting the PCC/PCUN to right PHG are negatively correlated with duration of heroin use is partially in accordance with published DTI studies in chronic HDIs [11,12]. This further supports our previous finding that the severity of WM integrity deficits in HDIs is associated with length of heroin dependence [11]. Functional connectivity of the PCC/PCUN and the MPFC and functional connectivity of the PCC/PCUN and the bilateral PHG tended to be correlated with duration of heroin use, though not significantly. Together with previous studies of HDIs [11,46,47], it is plausible that heroin abuse has cumulative effects on the human brain, not only disrupting the white matter tracts but also the functional integrity of the DMN.

At last, there is a positive correlation between FA values of cingulate, functional connectivity between the PCC/PCUN and MPFC and IGT performance. It is believed that the dorsal part of the ACC plays a key role in reward-based decisionmaking and learning [48]. The above positive correlation observed in present study further supports this hypothesis [48].

## Limitation

There were several limitations in this study. First, the small sample size in this study may have affected the statistical significance. Additional investigations involving larger sample sizesare needed to verify our results. Second, all of the subjects were inpatients and were receiving methadone at the time of the MRI study. MMT can impair white matter integrity [49] and influence resting state brain function [40]. Although no correlation between DMN structural and functional connectivity indices and methadone was found, the influence of methadone cannot be entirely excluded. Therefore, further studies on the influence of methadone on DMN structural and functional connectivity are needed. Another limitation was that the ROIs were selected on the DMN map from the controls. Nonetheless, possible differences in network topology between controls and HDIs should be considered. ROIs were chosen from the DMN map of controls since reduced connectivity in HDIs could have resulted in a poor estimation of ROI locations. The selection of the seed nodes in the network, as well as the seed size, is still matter of debate in functional connectivity studies [50,51]. It is important to consider that large ROIs can blur the results of functional connectivity analyses. In the current study, ROIs were defined that comprised more than one brain area, based on the ICA results, without using anatomical information or prior seed coordinates from the literature [52]. Lastly, in this study, the structural and subsequent functional connectivity analyses were restricted to the three pairs of DMN regions that showed fiber connection in all subjects. However, functional connectivity may exist between regions that do not show direct structural connectivity as detected by DTI. In the current study, only IGT tests were used. Although the IGT task has been widely used to assess the decision making in addicts [19–22], in the future, abroader spectrum of tests could be used to evaluate the cognitive function of HDIs.

## Conclusion

In summary, this study demonstrated both disturbed functional and structural connectivity within the DMN in HDIs. Functional and structural connectivity was also correlated with duration of heroin use and IGT performance in HDIs. The altered connectivity of the DMN may serve as a potential biomarker for evaluating HDIs.

## Supporting Information

S1 Fig When using the 24-parameter head motion model, we found similar results to those reported (A: DMN for HDIs, B: DMN for control, C: DMN for HDIs using 24-parameter head motion model, D: DMN for the control using 24-parameter head motion model). (TIF)

S2 Fig The DMN was similar to the controls when using a single ICA analysis with all 28 patients in one group (A and B is the DMN estimate for the heroin and control group individually; C represent DMN estimate by using a single ICA analysis). (TIF)
