# Supplementary figures and images for: Aberrant Default-Mode Functional and Structural Connectivity in Heroin-Dependent Individuals
Source: PLoS One. 2015 Apr 10;10(4):e0120861. doi: 10.1371/journal.pone.0120861 (PMC4393019; doi:10.1371/journal.pone.0120861)

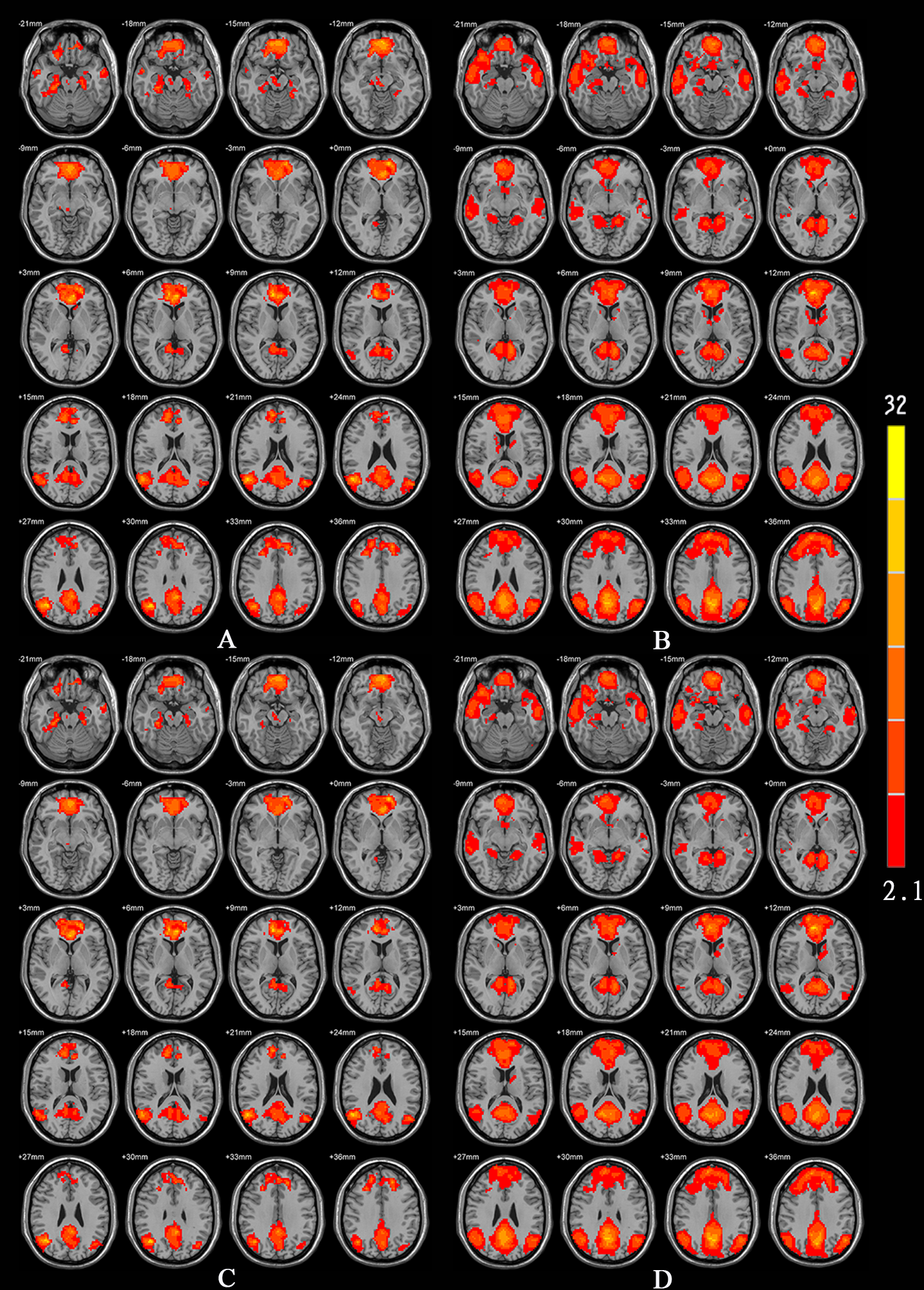

Supplement: S1 Fig — (TIF) [file pone.0120861.s001.tif]

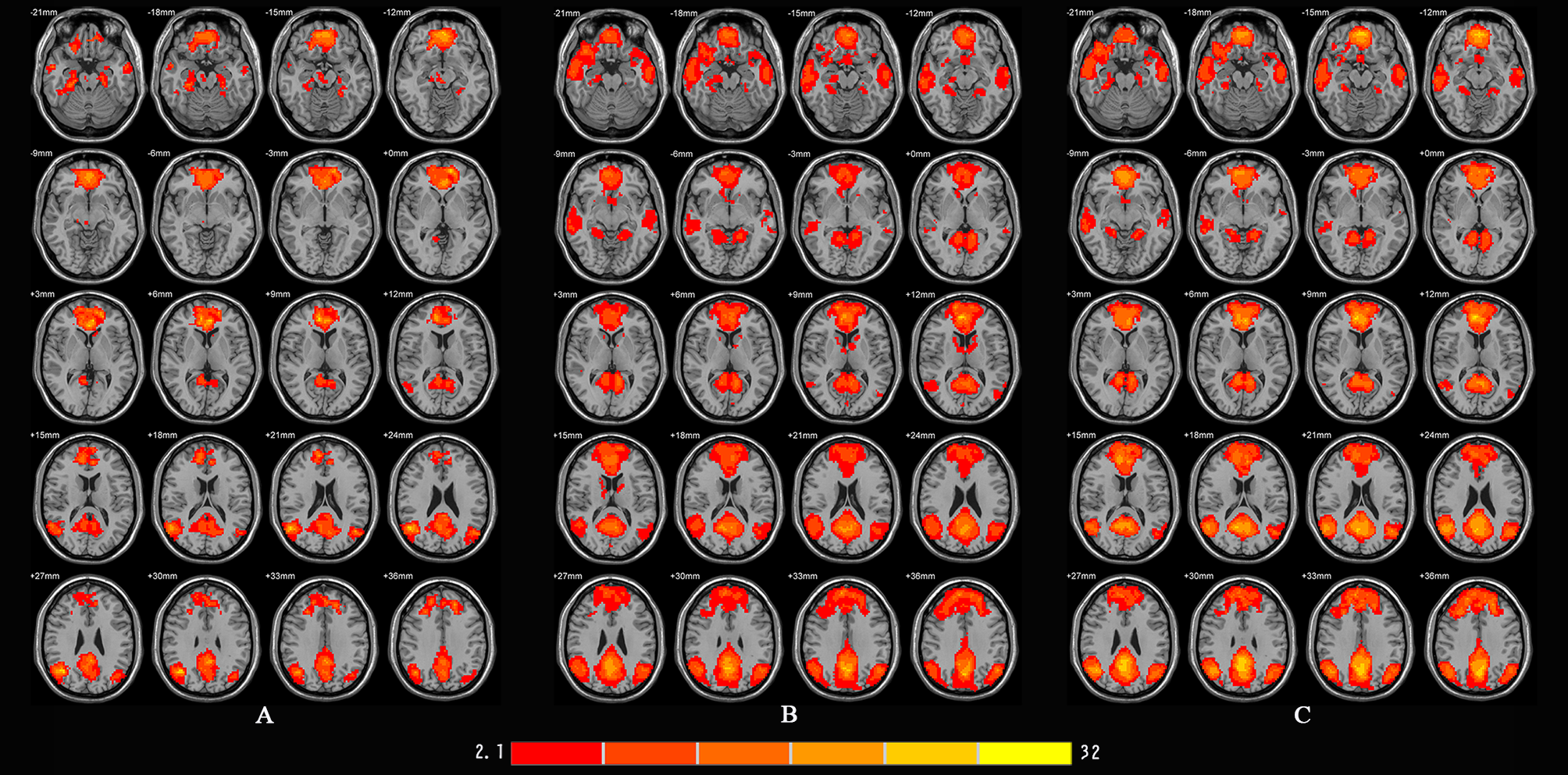

Supplement: S2 Fig — (TIF) [file pone.0120861.s002.tif]
